# Efficacy and safety of stem cell therapy for Crohn’s disease: a meta-analysis of randomized controlled trials

**DOI:** 10.1186/s13287-024-03637-z

**Published:** 2024-02-02

**Authors:** Yunfeng Qiu, Changfeng Li, Shihou Sheng

**Affiliations:** 1 Department of Endoscopy Center, China-Japan Union Hospital of Jilin University, Changchun, 130033 China; 2 Department of Gastrointestinal Surgery, China-Japan Union Hospital of Jilin University, No. 126, Xiantai Street, Changchun, 130033 China

**Keywords:** Crohn’s disease, Stem cell therapy, Clinical remission, Adverse events, Meta-analysis

## Abstract

**Purpose:**

Small-scale clinical trials have provided evidence suggesting the effectiveness of stem-cell therapy (SCT) for patients diagnosed with Crohn's disease (CD). The objective of the research was to systematically assess the effectiveness and safety of SCT for individuals diagnosed with CD through a comprehensive review and meta-analysis.

**Methods:**

A search was conducted in Medline (PubMed), CENTER (Cochrane Library), and Embase (Ovid) to find randomized controlled trials (RCTs) that assessed the impact of SCT on the occurrence of clinical remission (CR) and severe adverse events (SAE) among patients diagnosed with CD. The Cochrane Q test and estimation of *I*^2^ were used to assess heterogeneity among studies. After incorporating heterogeneity, a random-effects model was employed for data pooling.

**Results:**

Overall, 12 RCTs involving 632 adult patients with medically refractory CD or CD-related fistula were included. In comparison with placebo or no treatment, SCT showed a greater likelihood of CR (odds ratio [OR] 2.08, 95% CI 1.39–3.12, *p* < 0.001) without any notable heterogeneity (*I*^2^ = 0%). Consistent results were observed in subgroup analyses based on study design, patient diagnosis, source and type of stem cells, and follow-up durations, with all p-values for subgroup analyses being greater than 0.05. The occurrence of SAE was similar among patients assigned to SCT and the placebo/no treatment cohorts (OR 0.70, 95% CI 0.37–1.33, *p* = 0.28; *I*^2^ = 0%).

**Conclusions:**

For patients with medically refractory CD or CD-related fistula, SCT may be an alternatively effective and safe treatment.

**Supplementary Information:**

The online version contains supplementary material available at 10.1186/s13287-024-03637-z.

## Introduction

Crohn's disease (CD), classified as an inflammatory bowel disease, is a chronic systemic condition that is mediated by the immune system [1, 2]. It is characterized by inflammation in the gastrointestinal tract and dysregulation of the immune system associated with the mucosa [3]. The etiology of CD is evidently autoimmune in nature, as supported by its pathogenic mechanism [4, 5]. The clinical manifestation of this disease encompasses a range of symptoms including diarrhea, intestinal fistulas, strictures, malabsorption, and weight loss [6]. Studies have reported an annual incidence rate of CD ranging from 5.0 to 20.2 per 100,000 person-years [7]. Furthermore, recent research has indicated that hospitalization rates for CD are stabilizing in developed countries, while newly industrialized nations are experiencing a rapid increase in hospitalization rates [8]. This trend contributes to an escalating burden on global healthcare systems [8]. Presently, corticosteroids, immunomodulatory agents, and biological therapies specifically anti-tumor necrosis factor-alpha (TNFa) agents, are widely accepted conventional medical interventions employed to mitigate intestinal inflammation [9]. Nevertheless, approximately 25% of patients diagnosed with CD are resistant to these pharmaceutical interventions, thus being classified as having medically refractory CD [10]. Within this subgroup, perianal CD-related fistula denotes the ineffectiveness of both surgical therapeutic approaches and anti-TNFa agents [11]. In the realm of clinical practice, it is imperative to explore alternative treatments to enhance the likelihood of achieving clinical remission, particularly for those with medically refractory CD and complex CD-related fistula.

Stem cells have emerged as a novel approach for eliciting immunomodulatory effects and restoring immune homeostasis [12, 13]. Recent advancements in stem cell biology and regenerative medicine have unveiled their unforeseen potential in the treatment of autoimmune disorders [14]. Furthermore, stem cells possess regenerative properties that can facilitate tissue healing [15]. Accumulating evidence derived from preclinical studies and pilot clinical trials has demonstrated the potential of stem cell therapy (SCT) to impede intestinal inflammation, foster sustained healing of the intestinal mucosa, and substantially enhance the quality of life for patients [16, 17]. Consequently, SCT has emerged as a valuable alternative treatment for individuals with CD [18]. Nonetheless, it is important to note that the available evidence primarily stems from limited-scale clinical trials [19]. Consequently, the objective of this study was to conduct a systematic review and meta-analysis to comprehensively assess the effectiveness and safety of SCT in patients diagnosed with CD.

## Methods

This study is in accordance with the guidelines of Preferred Reporting Items for Systematic Reviews and Meta-Analyses (PRISMA) [20, 21] and Cochrane Handbook [22]. A filled PRISMA Checklist has been provided in Additional file 1: Material 1. The meta-analysis protocol was registered with the registration number of INPLASY2023100047 at the International Platform of Registered Systematic Review and Meta-analysis Protocols (INPLASY, https://inplasy.com/).

### Study inclusion and exclusion criteria

The principle of PICOS, which is explained below, was utilized to determine the inclusion criteria for the meta-analysis.

P (patients): Adult patients with CD who had already received standard medical treatments;

I (intervention): SCT, regardless the source, type, protocol, or session of the treatment;

C (control): Administration of a placebo or the absence of any treatment;

O (outcomes): The effectiveness measure was the occurrence of CR observed among patients assigned to the SCT and the control groups. The definition of CR was consistent with those used among the original studies. In general, patients with CD that does not respond to medical treatment are considered to have achieved CR if their Crohn's Disease Activity Index (CDAI) score is less than 150 [23]. Similarly, for patients with CD-related fistula, CR is defined as the complete healing of the fistula based on both clinical and radiological assessments [24]. The safety outcome was determined by the occurrence of severe adverse events (SAE), which encompassed events resulting in fatality or life-threatening situations, necessitating hospitalization or causing disability, or requiring an intervention to prevent any of these outcomes.

S (study design): Only RCTs with parallel groups that were published as complete articles in English in peer-reviewed journals were deemed eligible for study design.

The excluded studies include studies that were not limited to patients with CD, or studies that did not report the desired outcomes. In case studies with potentially overlapping patient populations were found, the meta-analysis included the one that had the larger sample size.

### Literature search strategy

To identify studies in Medline (PubMed), CENTER (Cochrane Library), and Embase (Ovid), a search strategy was employed that encompassed the following criteria: (1) 'stem cell' OR 'precursor cell' OR 'progenitor cell' OR 'stromal cell'; (2) 'inflammatory bowel disease' OR 'crohn’s disease' OR 'crohn disease' OR 'regional enteritis' OR 'ileocolitis' OR 'granulomatous colitis' OR 'granulomatous enteritis'; and (3) 'random' OR 'randomised' OR 'randomized' OR 'randomly' OR 'placebo' OR 'control'. A detailed search strategy is provided in Additional file 2: Material 2. Our focus was solely on research that involved human participants. In addition, we conducted a manual search for references to relevant reviews and primary articles. The most recent database search was conducted on September 05, 2023.

### Extraction of data and assessment of study quality

Two authors conducted separate searches in databases, gathered information, and assessed the quality. In case of any disagreements, the corresponding author was consulted to reach a consensus. For the study, various data was gathered including general details, characteristics of the study design, patient attributes such as diagnosis, sample size, age, gender, and background therapy. Additionally, details about interventions such as type, source, amount, protocol, and session of SCT, as well as information about controls (placebo or no additional treatment), follow-up durations, and the definition of CR were collected. The assessment of study quality was conducted utilizing the risk-of-bias assessment tool version 2.0 (ROB 2.0) as recommended by Cochrane [25]. This evaluation tool appraises the risk of bias across five distinct domains. In the event that the evaluation yields low risk in all five domains, the overall risk of bias is deemed low. Conversely, if any one domain is assessed as high risk or multiple domains indicate potential risk, the overall risk is considered high. Should neither of the aforementioned conditions be met, the clinical control trial is classified as having a potential risk of bias. In addition, the certainty of evidence was also evaluated using the GRADE (Grading of Recommendations, Assessment, Development and Evaluation) system, which includes risk of bias, inconsistency, indirectness, imprecision and publication bias [26]. The certainty of evidence was classified as very low, low, moderate or high.

### Statistical analysis

The odds ratio (OR) and the 95% confidence interval (CI) were used to summarize the impact of SCT on the likelihood of CR and occurrence of SAE in individuals diagnosed with CD. The Cochrane Q test was used to investigate the heterogeneity among the included studies [22]. Furthermore, the *I*^2^ statistic was calculated, where *I*^2^ > 50% suggests considerable heterogeneity [27]. Because of the anticipated heterogeneity in study designs, patient characteristics, and interventions, we used the random-effect model to pool the results by incorporating the potential influences of heterogeneity, using the DerSimonian and Laird estimator [22, 28]. Sensitivity analysis was performed to evaluate the influence of individual study on the outcome of the primary meta-analyses by excluding one study at a time [22]. Moreover, sensitivity analysis was also performed by excluding studies with high risk of bias as evaluate by ROB 2.0. Predefined subgroups were also analyzed based on predetermined study characteristics, including the study's design (open-label, single-blind, or double-blind), patient diagnosis (medically refractory CD or CD-related fistula), type of stem cells (derived from adipose tissue, bone marrow, or other sources), stem cell source (autologous or allogenic), and duration of follow-up (up to 12 weeks, 24 weeks, or 52 weeks). Publication bias was evaluated using Egger's test for regression asymmetry and funnel plots [29]. A p-value less than 0.05 suggest a statistically significant distinction. The statistical analysis was conducted using RevMan (Version 5.1; Cochrane, Oxford, UK) and Stata (Version 12.0; Stata Corporation, US) software.

## Results

### Literature search

**Fig. 1:**
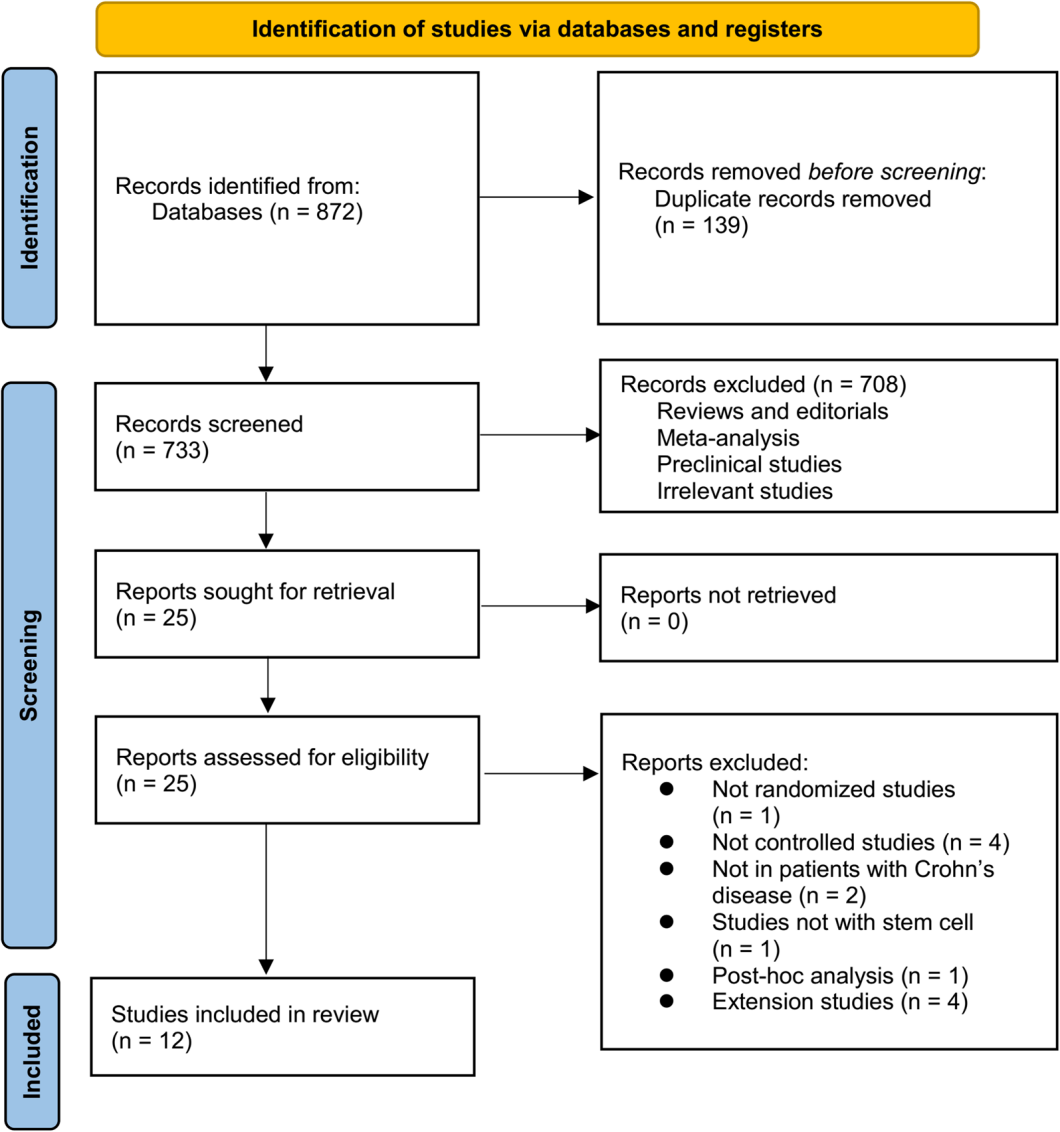
Flowchart of the literature search

The process of acquiring literature is illustrated in Fig. 1. In summary, a total of 872 articles were obtained through database searches, with 733 remaining after removing duplicates. A grand total of 708 articles were subsequently eliminated due to their titles and abstracts, primarily because they were not relevant to the objective of the study. After reading the full text, an additional 13 articles out of the initial 25 were excluded due to the reasons outlined in Fig. 1. At last, 12 RCTs [30–41] were available for the subsequent meta-analysis.

### Study characteristics and data quality evaluation

Table 1 provides a summary of the studies that are included in the meta-analysis. In total, there were 12 RCTs involving 632 adult individuals who had medically resistant CD or CD-associated fistula. These research papers were released from 2009 to 2023 and conducted in various locations including Spain [30], the Netherlands [33], the United States [32, 38–41], China [35, 36], Italy [37], and several centers across Europe and Israel [31, 34]. The sample size of the included study was 6–212. As for the diagnosis of the patients, four studies included patients with medically refractory CD [31, 32, 35, 38], while the other eight studies included patients with CD-related fistula [30, 33, 34, 36, 37, 39–41]. The mean age of the patients was 24.7–50.4 years, and the proportion of men was 0–95.5%. Standard medical therapy for CD has been used for all of the included patients, which included glucocorticoids, immunosuppressive agents, and anti-tumor necrosis factor alpha agents. Adipose-derived stem cells (ADSCs) were used as intervention in four studies [30, 34, 36, 37], bone marrow-derived mesenchymal stem cells (BM-MSCs) and hematopoietic stem cells (HSCs) were used in six studies [31, 33, 38–41], while in the other two studies, and placenta-derived cells [32] and umbilical cord mesenchymal stem cells (UC-MSCs) [35] were used, respectively. As for the source of stem cells, autologous stem cells were used in four studies [30, 31, 36, 37], while allogeneic stem cells were used in the other eight studies [32–35, 38–41]. The amount of stem cells used for each session varied among the included studies. For delivering of the stem cells, local injection was performed in nine studies [30, 33, 34, 36–41], while intravenous infusion was performed in three studies [31, 32, 35], including one or two sessions. The follow-up durations varied between 6 and 52 weeks. Table 2 provides a detailed analysis of the included RCTs using ROB 2.0. Five of the included studies were open-label [30, 31, 35–37], four were single-blind [38–41], while the other three were double-blind [32–34]. The randomization process was considered to be of some concerns in three studies because the details of allocation concealment were not adequately reported [32, 35, 38]. The measurement of the outcome was considered to be of high risk in seven studies [31, 35, 36, 38–41] largely because the outcome assessors were not blinded to the intervention. No concern of bias was detected in other domains such as deviations from intended interventions, missing outcome data, or selection of the reported result. Overall, four studies were judged to be of low risk of bias [30, 33, 34, 37], one was with some concerns [32], and another seven studies [31, 35, 36, 38–41] were of high risk of bias.

**Table 1:** Characteristics of the included RCTs

Study	Location	Design	Diagnosis	Patient number	Mean age (years)	Men (%)	Background therapy	Type and source of stem cells	Amount, delivery methods, and sessions of SCT	Control	Follow-up duration	Definition of CR
Garcia-Olmo 2009	Spain	R, OL	CD with complex perianal fistula	14	43.3	49	Standard medical management including immunomodulators and infliximab, antibiotics, and fibrin glue	ADSCs, autologous	1*10^7 cells, local injection, 1–2 sessions	No additional treatment	8 weeks	Absence of drainage through the external openings and complete reepithelialization of external openings
Molendijk 2015	The Netherlands	R, DB, PC	CD with refractory perianal fistulas	21	37.8	57.1	Mesalamine and steroids 4 weeks; immunosuppressive drugs 8 weeks; anti-TNFa agents 8 weeks	BM-MSCs, allogeneic	1*, 3*, or 9* 10^7 cells, local injection, 1 session	Placebo (solution with no cells)	12 weeks	Reduction in the number of draining fistulas determined by absence of discharge at physical examination and absence of collections of 2 cm directly related to the treated fistula tracts as measured by MRI
Melmed 2015	USA	R, DB, PC	Moderate-to-severe CD with CDAI: 220 ~ 450	46	35.7	47.1	Stable doses of immunomodulators and/or biologics	Placenta-derived cells, allogeneic	1.5* or 5* 10^8 cells, IV infusion, 1–2 sessions	Placebo (solution with no cells)	6 weeks	CDAI < 150
Hawkey 2015	11 European centers	R, OL	Medically refractory CD	45	32.4	46.7	Azathioprine/6 mercaptopurine, Methotrexate, and anti-TNFa agents	HSCs, autologous	9.0*10^6 cells/kg, IV infusion, 1 session	No additional treatment	52 weeks	CDAI < 150
Panes 2016	49 European and Israel centers	R, DB, PC	CD with complex perianal fistula	212	38.3	54.8	Immunomodulators and anti-TNFa agent	Adipose-derived MSCs, allogenic	12*10^7 cells, local injection, 1–2 sessions	Placebo (solution with no cells)	24 weeks	Clinical assessment of closure of all treated external openings that were draining at baseline, and the absence of collections larger than 2 cm of the treated perianal fistulas in at least two of three dimensions, confirmed by masked central MRI
Zhang 2018	China	R, OL	Medically refractory CD	82	33.5	61	Glucocorticoids, immunosuppressive agents, and anti-TNFa agent	UC-MSCs, allogenic	1*10^6 cells/kg, IV infusion, 4 sessions	No additional treatment	52 weeks	CDAI < 150
Zhou 2020	China	R, OL	CD with complex perianal fistula	22	24.7	95.5	ASA, probiotics, immunomodulators, antibiotics, glucocorticoids, and anti-TNFa agent	ADSCs, autologous	1*10^7 cells, local injection, 1 sessions	No additional treatment	52 weeks	Complete epithelialization of external openings (i.e., no pus outflow from the external openings under any circumstances) and no evidence of fistulas in MRI or ERUS
Ascanelli 2021	Italy	R, OL	CD with complex perianal fistula	120	50.4	61.2	Standard medical treatments (not specified)	ADSCs, autologous	Cells in 16 ml adipose tissue, local injection, 1–2 sessions	No additional treatment	24 weeks	External opening was closed with no perianal discharge on clinical assessment and MRI
Lightner 2022	USA	R, SB, PC	Refractory CD	6	41.6	66.7	Standard medical treatments (not specified)	BM-MSCs, allogeneic	15* or 30* 10^7 cells, local injection, 1–2 sessions	Placebo (saline)	12 weeks	CR*p* < 2.87 mg/l and CDAI < 150
Lightner 2023a	USA	R, SB, PC	CD with complex perianal fistula	23	33.9	47.8	Glucocorticoids, immunosuppressive agents, and anti-TNFa agent	BM-MSCs, allogeneic	7.5* 10^7 cells, local injection, 1–2 sessions	Placebo (saline)	24 weeks	Complete cessation in drainage and the external opening was epithelialized on clinical examination and an absence of a fluid collection ≥ 2 cm in any 2 of 3 dimensions and a lack of edema, inflammation, or sign of active inflammatory response on MRI
Lightner 2023b	USA	R, SB, PC	CD with peripouch fistulas	22	41.2	31.8	Glucocorticoids, immunosuppressive agents, and anti-TNFa agent	BM-MSCs, allogeneic	7.5* 10^7 cells, local injection, 1 session	Placebo (saline)	24 weeks	External opening was closed with no discharge on clinical assessment and MRI
Lightner 2023c	USA	R, SB, PC	CD with rectovaginal fistulizing	19	42.6	0	Glucocorticoids, immunosuppressive agents, and anti-TNFa agent	BM-MSCs, allogeneic	7.5* 10^7 cells, local injection, 1–2 sessions	Placebo (saline)	24 weeks	Combined clinical and radiological healing of the rectovaginal fistulizing

RCTs, randomized controlled trials; SCT, stem cell therapy; CR, clinical remission; R, randomized; OL, open-label; SB, single-blind; DB, double-blind; PC, placebo-controlled; TNFa, tumor necrosis factor alpha; CD, Crohn’s disease; CDAI, Crohn's Disease Activity Index; ADSCs, adipose-derived stem cells; BM-MSCs, bone marrow-derived mesenchymal stem cells; HSCs, hematopoietic stem cells; MSCs, mesenchymal stem cells; UC-MSCs, umbilical cord mesenchymal stem cells; CRP, C-reactive protein; MRI, magnetic resonance imaging;

**Table 2:** Quality evaluation of the included studies with ROB 2.0

Study	Randomization process	Deviations from intended interventions	Missing outcome data	Measurement of the outcome	Selection of the reported result	Overall bias
Garcia-Olmo 2009	Low risk	Low risk	Low risk	Low risk	Low risk	Low risk
Molendijk 2015	Low risk	Low risk	Low risk	Low risk	Low risk	Low risk
Melmed 2015	Some concerns	Low risk	Low risk	Low risk	Low risk	Some concerns
Hawkey 2015	Low risk	Low risk	Low risk	High risk	Low risk	High risk
Panés 2016	Low risk	Low risk	Low risk	Low risk	Low risk	Low risk
Zhang 2018	Some concerns	Low risk	Low risk	High risk	Low risk	High risk
Zhou 2020	Low risk	Low risk	Low risk	High risk	Low risk	High risk
Ascanelli 2021	Low risk	Low risk	Low risk	Low risk	Low risk	Low risk
Lightner 2022	Some concerns	Low risk	Low risk	High risk	Low risk	High risk
Lightner 2023a	Low risk	Low risk	Low risk	High risk	Low risk	High risk
Lightner 2023b	Low risk	Low risk	Low risk	High risk	Low risk	High risk
Lightner 2023c	Low risk	Low risk	Low risk	High risk	Low risk	High risk

### Efficacy outcome: possibility of CR

**Fig. 2:**
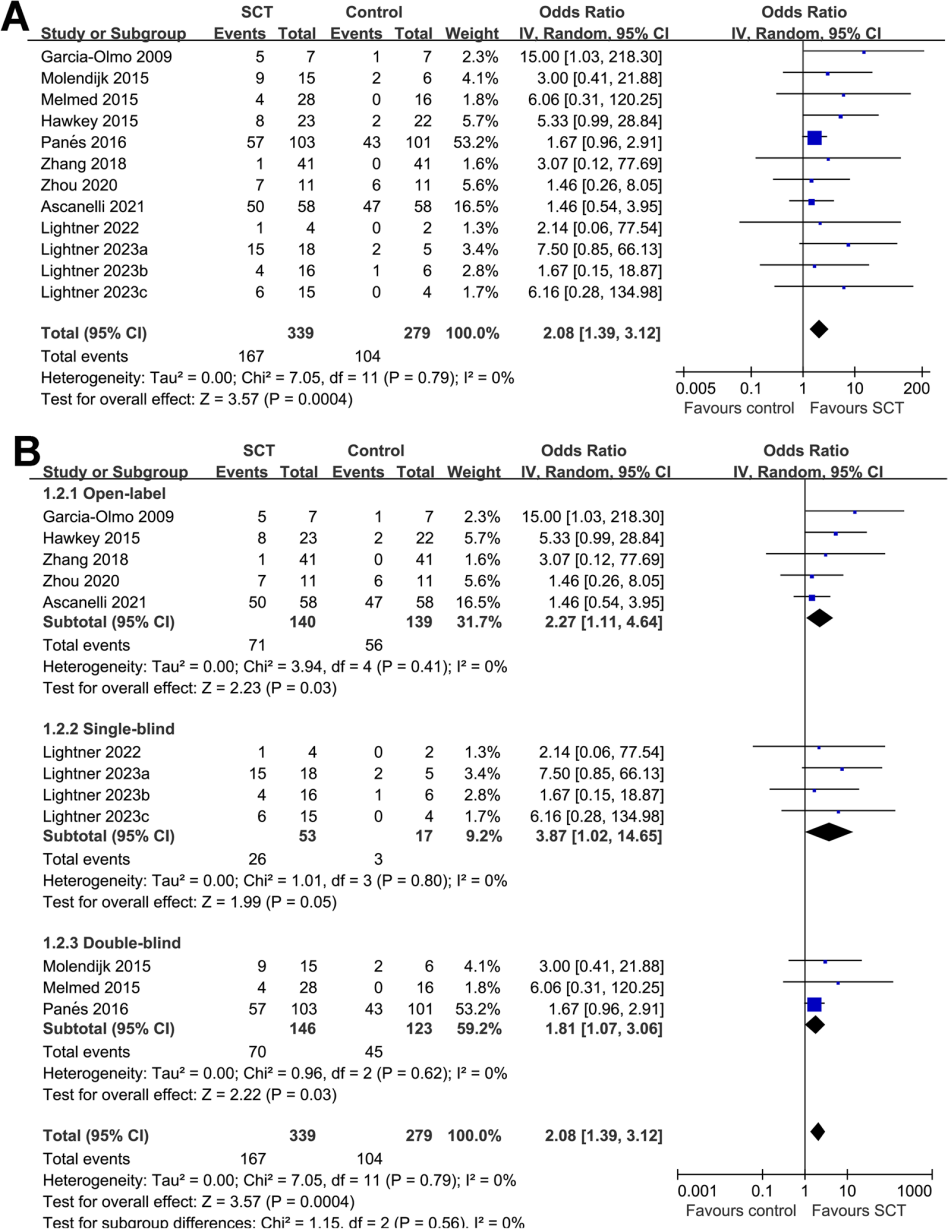
Forest plots for the meta-analysis evaluating the influence of SCT on CR in patients with CD. **A** Funnel plots for the overall meta-analysis; and **B** funnel plots for the subgroup analysis according to study design

**Fig. 3:**
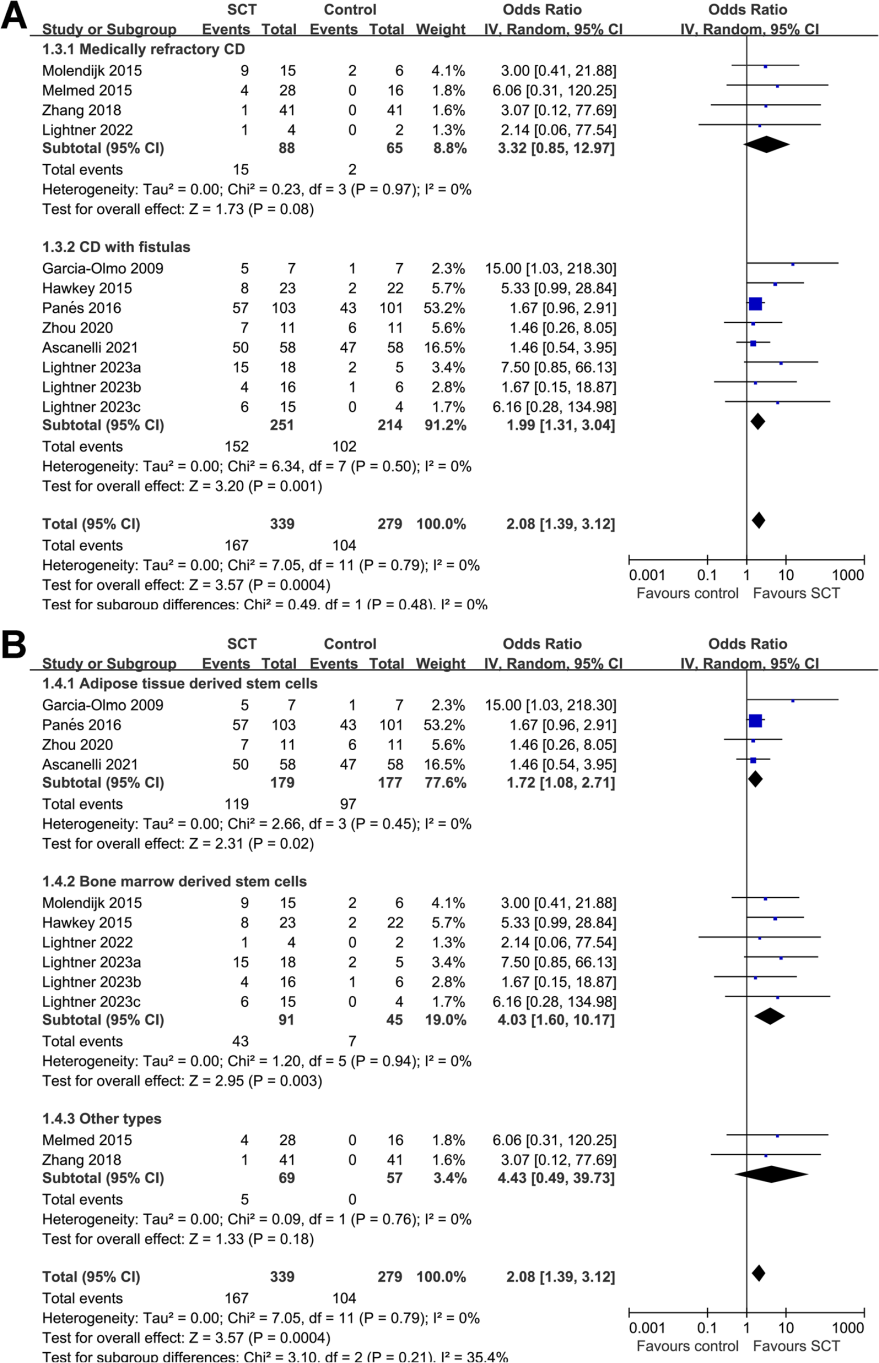
Forest plots for the subgroup analysis evaluating the influence of SCT on the possibility of CR in patients with CD. **A** Funnel plots for the subgroup analysis according to the diagnosis of the patients; and **B** funnel plots for the subgroup analysis according to type of stem cells used

**Fig. 4:**
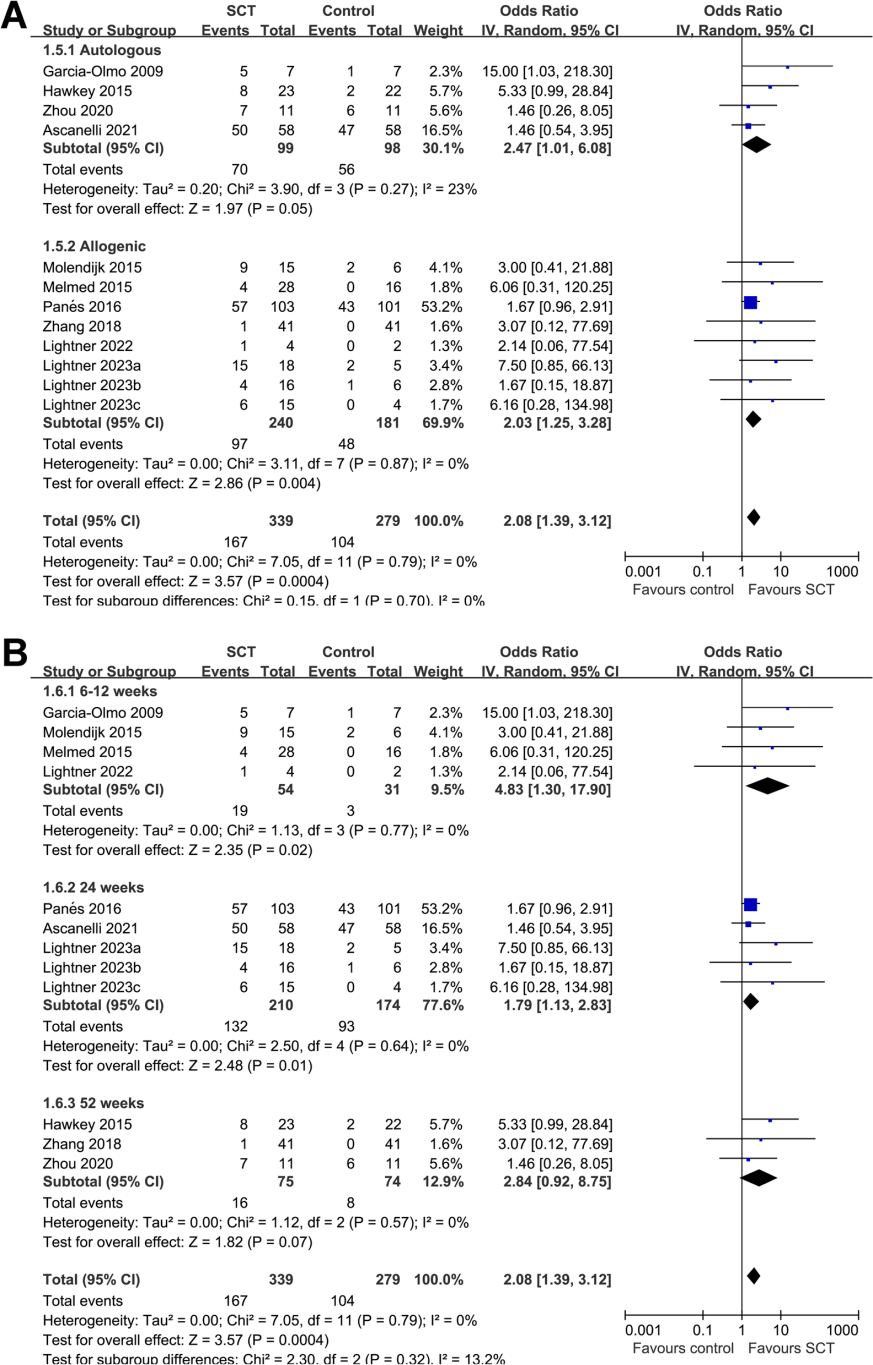
Forest plots for the subgroup analysis evaluating the influence of SCT on CR in patients with CD. **A** Funnel plots for the subgroup analysis according to the source of stem cells used; and **B** funnel plots for the subgroup analysis according to follow-up durations

Overall, 12 RCTs [30–41] reported the outcome of CR. Compared to placebo/no treatment, SCT was associated with a higher possibility of CR (OR 2.08, 95% CI 1.39–3.12, *p* < 0.001) in patients with CD with no significant heterogeneity (*I*^2^ = 0%; Fig. 2A). Sensitivity analysis by excluding one study at a time showed consistent results (OR 1.99–2.68, *p* all < 0.05; Additional file 3: Table 1). Moreover, sensitivity analysis by excluding studies of high risk of bias showed similar results (OR 1.84, 95% CI 1.17–2.91, *p* = 0.009; *I*^2^ = 0%). Subgroup analyses according to study design (Fig. 2B), diagnosis of the patients (Fig. 3A), type of stem cells (Fig. 3B), source of stem cells (Fig. 4A) and follow-up durations (Fig. 4B) showed consistent results (*p* for subgroup analyses all > 0.05). Summarized certainty of evidence using the GRADE system is shown in Table 3. We downgraded evidence by one level for the inadequate description of allocation concealment in three studies [32, 35, 38] and the lack of blinding in outcome assessment in seven studies [31, 35, 36, 38–41]. We judged the evidence to be of moderate certainty.

**Table 3:** Summarized certainty of evidence using the GRADE system

Outcome	Quality assessment							Absolute effect OR (95% CI)	Quality
	No. of studies	Design	Risk of bias	Inconsistency	Indirectness	Imprecision	Other considerations		
CR	12	RCTs	Serious	No serious inconsistency	No serious indirectness	No serious imprecision	None	2.08 (1.39–3.12)	⊕ ⊕ ⊕ Ο MODERATE
SAE	9	RCTs	Serious	No serious inconsistency	No serious indirectness	No serious imprecision	None	0.70 (0.37–1.33)	⊕ ⊕ ⊕ Ο MODERATE

GRADE, Grading of Recommendations, Assessment, Development and Evaluation; CR, clinical remission; SAE, severe adverse events; RCT, randomized controlled trials; OR, odds ratio; CI, confidence interval;

### Safety outcome: incidence of SAE

**Fig. 5:**
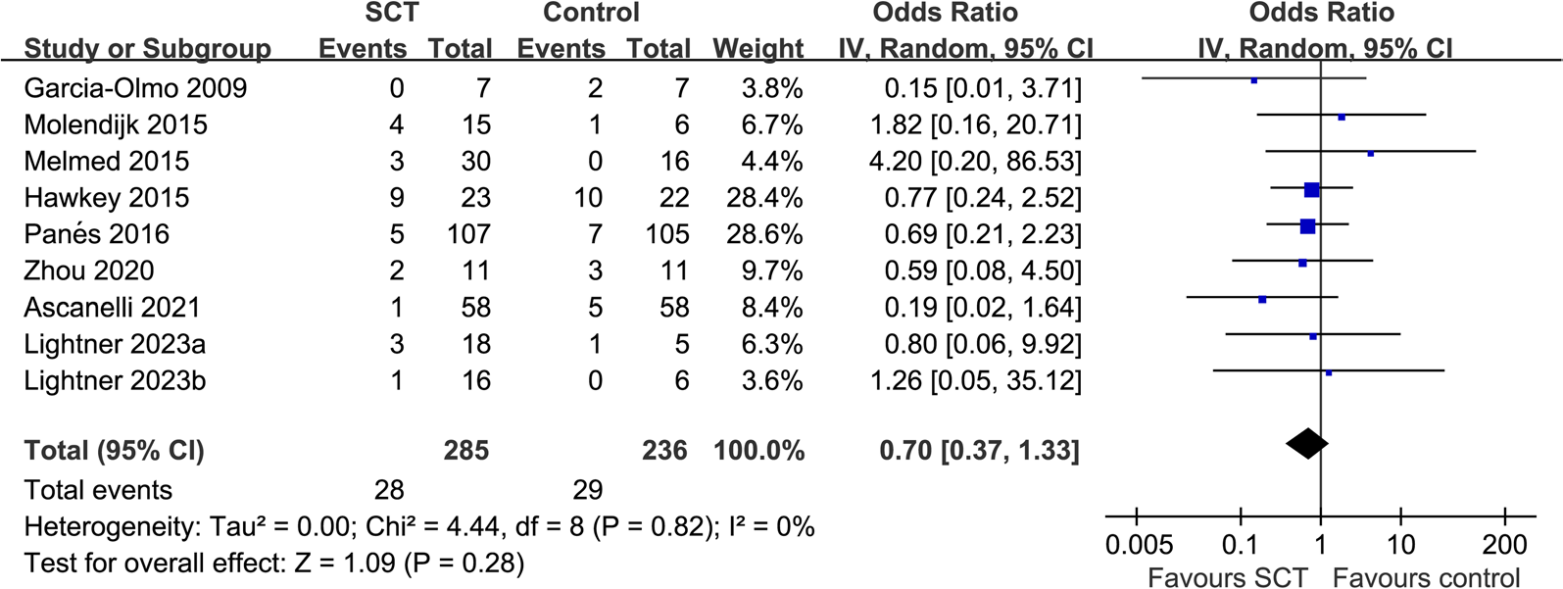
Forest plots for the meta-analysis evaluating the influence of SCT on the incidence of SAE in patients with CD

Three of the included studies did not report any SAE of patients in both groups [35, 38, 41]. Types of SAE reported in the nine studies are shown in Additional file 4: Table 2. Pooled results of the other nine studies [30–34, 36, 37, 39, 40] suggested that the incidence of SAE was comparable between patients allocated to SCT and the placebo/no treatment groups (OR 0.70, 95% CI 0.37–1.33, *p* = 0.28; *I*^2^ = 0%; Fig. 5). Sensitivity analysis by excluding one study at a time did not significantly affect the results (OR 0.65–0.80, p all > 0.05; Additional file 5: Table 3). In addition, sensitivity analyses by excluding studies of high risk of bias also showed similar results (OR 0.65, 95% CI 0.26–1.66, *p* = 0.37; *I*^2^ = 6%). As shown in Table 3, the evidence was downgraded by one level for the inadequate description of allocation concealment in one study [32] and the lack of blinding in outcome assessment in four studies [31, 36, 39, 40]. We judged the evidence to be of moderate certainty.

### Publication bias

**Fig. 6:**
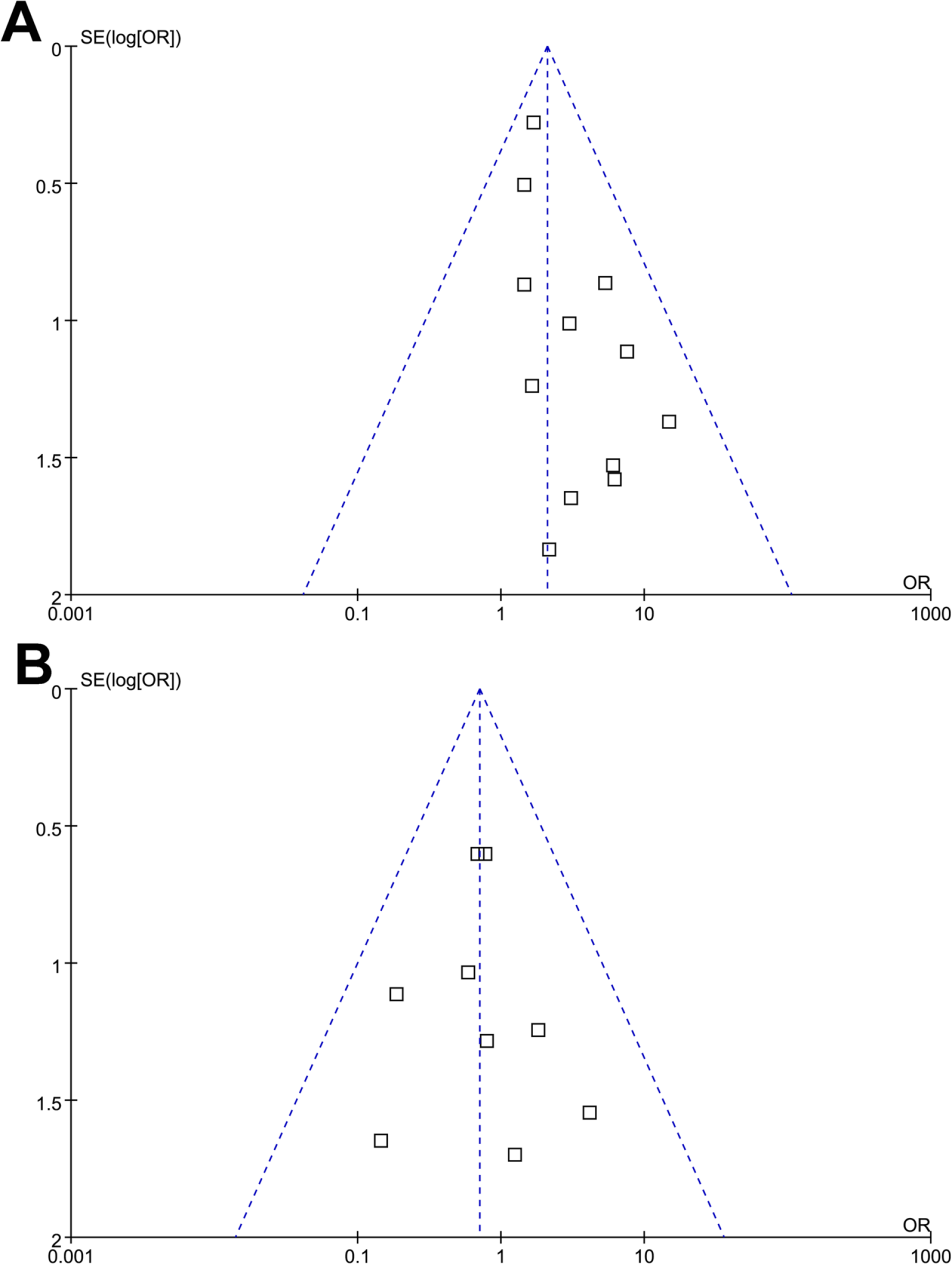
Funnel plots for the meta-analysis evaluating the influence of SCT on efficacy and safety outcomes in patients with CD. **A** Funnel plots for the meta-analysis of the influence of SCT on the possibility of CR; and **B** funnel plots for the meta-analysis of the influence of SCT on the incidence of SAE

Figure 6A, B shows symmetrical funnel plots in the meta-analyses of SCT effects on CR possibility and SAE incidence in CD patients, indicating a minimal risk of publication bias. Consistent results were also demonstrated by Egger's regression tests (*p* = 0.28 and 0.66, respectively).

## Discussion

In this study, by pooling the results of 12 pilot RCTs, the results of the meta-analysis showed that compared to controls with placebo/no additional treatment, SCT could significantly increase the possibility of CR in patients with medically refractory CD or CD-related fistula. Subsequent subgroup analyses showed that the benefits of SCT on CR in these patients were not significantly affected by predefined study characteristics such as study design, diagnosis of the patients, type and source of stem cells, or follow-up durations. Moreover, SCT was not associated with a significantly increased incidence of SAE. Taken together, these results indicate that SCT may be an alternatively effective and safe treatment for adult patients with medically refractory CD or CD-related fistula.

To the best of our knowledge, a few meta-analyses have been published previously to evaluate the efficacy and safety of SCT in patients with CD. An early meta-analysis in 2017 involving 21 studies comprising 514 patients with active CD showed that SCT may be effective to achieve a clinical response of 56% and a CR of 46%. However, the results were based mainly on single-arm observational studies without controls, and interpretation of the results in clinical setting is difficult [42]. Similar results were obtained in a meta-analysis in 2021, which showed that SCT may be effective in improving CDAI, rate of CR, endoscopic scores, and quality of life in patients with CD. However, the results were also obtained by summarizing single-arm observational studies [43]. Another meta-analysis included seven RCTs published up to 2020 suggested that SCT might be effective to achieve higher possibility of early CR as compared to controls, while the pooled results were not significant (risk ratio: 1.88, 95% CI 0.80–4.41), and the authors concluded uncertainty for the efficacy of SCT in patients with refractory CD [44]. A few strengths should be noticed in our meta-analysis as compared to the previous one. First, only RCTs were included in this study, which therefore minimized the confounding effect of potential imbalanced study characteristics between patients allocated to intervention and control groups on the outcome of the meta-analysis. Second, an updated literature search retrieved 12 eligible RCTs involving 632 adult patients with medically refractory CD or CD-related fistula. The sample size of the current meta-analysis is significantly larger than the previous one, and the pooled results confirmed a significant benefit of SCT on CR as compared to placebo/no additional treatment. Finally, a series of subgroup analyses were performed to evaluate the potential influences of predefined study characteristics on the efficacy of SCT on medically refractory CD, The results showed that the efficacy of SCT was not significantly affected by study design, diagnosis of the patients, source and type of stem cells, and follow-up duration, which further indicated the robustness of the finding. Taken together, these findings indicate SCT may be an alternatively effective and safe therapeutic option for patients with medically refractory CD. The results are of importance considering the limited treatments for this patient group.

The precise mechanisms responsible for the therapeutic effectiveness of SCT for CD have yet to be fully elucidated. However, several hypotheses can be proposed to elucidate the potential advantages of SCT for CD patients. Stem cells are known to be recruited to the damaged epithelial lining of the colon, where they subsequently differentiate into the necessary local cells for regenerating the affected mucosa or immune-modulatory cells. Consequently, stem cells are considered highly promising entities for restoring the barrier integrity of CD patients [45, 46]. Furthermore, it is of significant importance to note that stem cells have the potential to reinstate immune homeostasis through the transformation of detrimental pro-inflammatory responses into advantageous anti-inflammatory reactions [47]. For instance, the migration of mesenchymal stem cells (MSCs) to the site of disease activation, the secretion of various bioactive mediators and immunomodulatory molecules, as well as the recruitment of Treg cells and the redirection of T cell population toward a more regulatory phenotype, have been proposed as mechanisms through which MSCs can confer benefits on inflammatory bowel conditions, thereby exerting immunomodulatory effects [48, 49]. In a manner akin to MSCs, ADSCs have also demonstrated anti-inflammatory properties through the secretion of specific cytokines and extracellular vesicles, along with immunomodulation [50–52]. Nevertheless, the precise mechanisms and crucial molecular signaling pathways underlying the therapeutic advantages of SCT for CD necessitate further investigation in forthcoming research endeavors.

This study has several limitations. First, the definition of CR varied among the included studies. For patients with medically refractory CD, CR was defined by CDAI < 150; for patients with CD-related complex fistula, CR was defined by combined healing of fistula based on clinical and MRI evaluation. However, subgroup analysis according to the diagnosis of the patients showed consistent result, which suggested consistent benefit of SCT on CR in both patient groups. Second, the protocol of SCT varied among the included studies, such as the amount, delivery methods, and session of SCT. Although subgroup analysis according to type and source of stem cells showed similar results, these results should be interpreted with caution because the number of the studies included in each subgroup was limited. In addition, the dose of the stem cell may affect the efficacy of SCT for CD. However, the interaction between the dose of stem cells and the efficacy of SCT for CD could be significantly modified by multiple factors, such as type of stem cells, routes of administration, and sessions of SCT therapy. Unfortunately, our meta-analysis is based on the data of study level and unable to address these interactions. Studies are needed in the future to determine the optimal source, dose, routes of administration, and sessions of SCT therapy for CD. Moreover, five open clinical trials were included in the meta-analysis, which may introduce the potential risk of bias. However, the results of subgroup analysis for the outcome of CR were similar among open-label, single-blind, and double-blind studies. In addition, results of the meta-analysis were based on data of study level rather than data of individual patient level. Therefore, we were unable to determine the influence of patient characteristics on the outcome, such as age, sex, duration of the disease, disease activity at baseline, and concurrent treatment. Further investigation into these issues may be important for selecting the optimal candidates for SCT among patients with refractory CD. Moreover, we focused on the influence of SCT on the incidence of CR during follow-up rather than the maintenance of CR, and large-scale RCTs are needed in this regard. Finally, the follow-up duration of the studies included in the meta-analysis was up to 52 weeks. Although several extension studies have showing promising results [53, 54], the long-term efficacy and safety of SCT in patients with CD remain to be investigated in future studies.

## Conclusions

To sum up, the findings of the meta-analysis indicate that SCT may enhance the likelihood of achieving CR in patients suffering from medically resistant CD or CD-associated fistula. Furthermore, there was no correlation between SCT and a higher likelihood of SAE when compared to placebo or no supplementary intervention, even when observed for a period of up to 52 weeks. The results indicate that SCT could potentially serve as a viable and secure alternative therapy for individuals suffering from medically resistant CD. Further research is necessary to establish the most effective procedure of SCT for individuals with CD, and to assess the long-term effectiveness and safety of this therapy.

### Supplementary Information

**Additional file 1**. PRISMA Checklist.

**Additional file 2**. Detailed search strategy.

**Additional file 3**. Results of sensitivity analysis for the outcome of CR.

**Additional file 4**. Details of SAE in each study.

**Additional file 5**. Results of sensitivity analysis for the outcome of SAE.

## Data Availability

Data sharing is not applicable to this article as no datasets were generated or analyzed during the current study.
