# Supplementary material for: Efficacy and safety of stem cell therapy for Crohn’s disease: a meta-analysis of randomized controlled trials
Source: Stem Cell Res Ther. 2024 Feb 2;15:28. doi: 10.1186/s13287-024-03637-z (PMC10835827; doi:10.1186/s13287-024-03637-z)
Supplement: Supplementary file 2 — Additional file 2. Detailed search strategy. [file 13287_2024_3637_MOESM2_ESM.docx]

**Detailed search strategy**

**MEDLINE search strategy**

1. Crohn Disease/

2. Inflammatory Bowel Diseases/

3. (Crohn* or ileitis or regional enteritis or ileocolitis or granulomatous colitis or granulomatous enteritis).tw,kw.

4. (Inflammatory bowel disease* or IBD).tw,kw.

5. or/1-4

6. exp Stem Cell Transplantation/

7. Stem cell*.tw,kw.

8. SCT.tw,kw.

9. exp Stem Cells/

10. exp Hematopoietic Stem Cells/

11. ((autologous or autotransfusion or auto-transfus* or autograft* or allogenic) adj3 (hematopoietic or haematopoietic)).tw,kw.

12. exp Bone Marrow Transplantation/

13. (bone marrow adj3 (transplant* or graF* or transfus*)).tw,kw.

14. BMT.tw,kw.

15. ((autologous or autotransfusion or auto-transfus* or autograF* or allogenic) adj3 bone marrow).tw,kw.

16. or/6-15

17. 5 and 16

18. randomized controlled trial.pt.

19. controlled clinical trial.pt.

20. randomi?ed.ab.

21. placebo.ab.

22. drug therapy.fs.

23. randomly.ab.

24. trial.ab.

25. groups.ab.

26. or/18-25

27. exp animals/ not humans/

28. 26 not 27

29. 17 and 28

Limited to “clinical trial” or “randomized clinical trial”

**Cochrane CENTRAL search strategy**

1. Crohn Disease/

2. Inflammatory Bowel Diseases/

3. (Crohn* or ileitis or regional enteritis or ileocolitis or granulomatous colitis or granulomatous enteritis).tw,kw.

4. (Inflammatory bowel disease* or IBD).tw,kw.

5. or/1-4

6. exp Stem Cell Transplantation/

7. Stem cell*.tw,kw.

8. SCT.tw,kw.

9. exp Stem Cells/

10.exp Hematopoietic Stem Cells/

11. ((autologous or autotransfusion or auto-transfus* or autograft* or allogenic) adj3 (hematopoietic or haematopoietic)).tw,kw.

12. exp Bone Marrow Transplantation/

13. (bone marrow adj3 (transplant* or graF* or transfus*)).tw,kw.

14. BMT.tw,kw.

15. ((autologous or autotransfusion or auto-transfus* or autograF* or allogenic) adj3 bone marrow).tw,kw.

16. or/6-15

17. 5 and 16

**Embase search strategy**

1. Crohn disease/

2. inflammatory bowel disease/

3. (Crohn* or ileitis or regional enteritis or ileocolitis or granulomatous colitis or granulomatous enteritis).tw,kw.

4. (Inflammatory bowel disease* or IBD).tw,kw.

5. or/1-4

6. exp stem cell transplantation/

7. Stem cell*.tw,kw.

8. SCT.tw,kw.

9. exp stem cell/

10. ((autologous or autotransfusion or auto-transfus* or autograft* or allogenic) adj3 (hematopoietic or haematopoietic)).tw,kw.

11. exp bone marrow transplantation/

12. (bone marrow adj3 (transplant* or graF* or transfus*)).tw,kw.

13. BMT.tw,kw.

14. ((autologous or autotransfusion or auto-transfus* or autograF* or allogenic) adj3 bone marrow).tw,kw.

15. or/6-14

16. 5 and 15

17. random:.tw.

18. placebo:.mp.

19. double-blind:.tw.

20. or/17-19

21. exp animal/ not human/

22. 20 not 21

23. 16 and 22
