# Supplementary material for: Efficacy and safety of stem cell therapy for Crohn’s disease: a meta-analysis of randomized controlled trials
Source: Stem Cell Res Ther. 2024 Feb 2;15:28. doi: 10.1186/s13287-024-03637-z (PMC10835827; doi:10.1186/s13287-024-03637-z)
Supplement: Supplementary file 3 — Additional file 3. Results of sensitivity analysis for the outcome of CR. [file 13287_2024_3637_MOESM3_ESM.docx]

**Supplemental table 1** Results of sensitivity analysis for the outcome of CR

| Study omitted | OR (95% CI) | P for effect | I^2^ |
| --- | --- | --- | --- |
| Garcia-Olmo 2009 | 1.99 [1.32, 3.00] | 0.001 | 0% |
| Molendijk 2015 | 2.05 [1.36, 3.10] | < 0.001 | 0% |
| Melmed 2015 | 2.04 [1.36, 3.07] | < 0.001 | 0% |
| Hawkey 2015 | 1.97 [1.30, 2.98] | 0.001 | 0% |
| Panes 2016 | 2.68 [1.49, 4.84] | 0.001 | 0% |
| Zhang 2018 | 2.07 [1.38, 3.11] | < 0.001 | 0% |
| Zhou 2020 | 2.13 [1.41, 3.23] | < 0.001 | 0% |
| Ascanelli 2021 | 2.24 [1.44, 3.48] | < 0.001 | 0% |
| Lightner 2022 | 2.08 [1.39, 3.13] | < 0.001 | 0% |
| Lightner 2023a | 1.99 [1.32, 3.00] | 0.001 | 0% |
| Lightner 2023b | 2.10 [1.39, 3.16] | < 0.001 | 0% |
| Lightner 2023c | 2.05 [1.36, 3.07] | < 0.001 | 0% |

CR, clinical remission; OR, odds ratio; CI, confidence interval;
