# Supplementary material for: Efficacy and safety of stem cell therapy for Crohn’s disease: a meta-analysis of randomized controlled trials
Source: Stem Cell Res Ther. 2024 Feb 2;15:28. doi: 10.1186/s13287-024-03637-z (PMC10835827; doi:10.1186/s13287-024-03637-z)
Supplement: Supplementary file 4 — Additional file 4. Details of SAE in each study. [file 13287_2024_3637_MOESM4_ESM.docx]

**Supplemental table 2** Details of SAE in each study

| Study omitted | SCT | Control |
| --- | --- | --- |
| Garcia-Olmo 2009 | None; | Crohn’s crisis and intra-abdominal abscess in 1 patients; Perianal abscess in 1 patients; |
| Molendijk 2015 | Perianal abscess in 3 patients; adenocarcinoma of the cecum in 1 patient; | Perianal abscess in 1 patient; |
| Melmed 2015 | Hypersensitivity reaction in 1 patient; gastric ulcer perforation in 1 patient; anal cancer in 1 patient; | None; |
| Hawkey 2015 | Severe infection in 4 patients; disease flare in 3 patients; non-flare gastroenterological symptom in 1 patient; and severe anemia in 1 patient | Severe infection in 2 patients; disease flare in 7 patients; non-flare gastroenterological symptom in 1 patient; |
| Panes 2016 | Perianal abscess in 5 patients; | Perianal abscess in 5 patients; Proctalgia in 1 patient; Anal inflammation in 1 patient; Liver abscess in 1 patient; |
| Zhou 2020 | Perianal abscess in 2 patients; | Perianal abscess in 3 patients; |
| Ascanelli 2021 | Perianal abscess in 1 patient; | Perianal abscess in 4 patients; Bleeding in 1 patient; |
| Lightner 2023a | Anal abscess in 3 patients; | Anal abscess in 1 patient; |
| Lightner 2023b | Anal abscess in 1 patient; | None; |

SAE, severe adverse events; SCT, stem cell therapy;
