# Supplementary material for: Efficacy and safety of stem cell therapy for Crohn’s disease: a meta-analysis of randomized controlled trials
Source: Stem Cell Res Ther. 2024 Feb 2;15:28. doi: 10.1186/s13287-024-03637-z (PMC10835827; doi:10.1186/s13287-024-03637-z)
Supplement: Supplementary file 5 — Additional file 5. Results of sensitivity analysis for the outcome of SAE. [file 13287_2024_3637_MOESM5_ESM.docx]

**Supplemental table 3** Results of sensitivity analysis for the outcome of SAE

| Study omitted | OR (95% CI) | P for effect | I^2^ |
| --- | --- | --- | --- |
| Garcia-Olmo 2009 | 0.75 [0.39, 1.43] | 0.38 | 0% |
| Molendijk 2015 | 0.66 [0.34, 1.27] | 0.21 | 0% |
| Melmed 2015 | 0.65 [0.34, 1.24] | 0.19 | 0% |
| Hawkey 2015 | 0.68 [0.32, 1.43] | 0.31 | 0% |
| Panes 2016 | 0.71 [0.34, 1.50] | 0.37 | 0% |
| Zhou 2020 | 0.72 [0.37, 1.40] | 0.33 | 0% |
| Ascanelli 2021 | 0.80 [0.41, 1.54] | 0.50 | 0% |
| Lightner 2023a | 0.70 [0.36, 1.34] | 0.28 | 0% |
| Lightner 2023b | 0.69 [0.36, 1.31] | 0.26 | 0% |

SAE, severe adverse events; OR, odds ratio; CI, confidence interval;
